# Association of Biomarkers of Inflammation with Dyslipidemia and Its Components among Mongolians in China

**DOI:** 10.1371/journal.pone.0089023

**Published:** 2014-02-18

**Authors:** Lingyan Tang, Hao Peng, Tian Xu, Aili Wang, Guiyan Wang, Weijun Tong, Yonghong Zhang

**Affiliations:** 1 Department of Epidemiology, School of Public Health, Medical College of Soochow University, Suzhou, Jiangsu, China; 2 Department of Health Care, Xiamen Maternal and Child Care Hospital, Xiamen, Fujian, China; University of Leicester, United Kingdom

## Abstract

**Objective:**

This study aims to examine the association between inflammatory biomarkers and dyslipidemia and its components among Mongolians in China.

**Methods:**

Data were obtained from 2544 Mongolians via standard questionnaires and blood samples in Inner Mongolia, China. High sensitivity C-reactive protein (hsCRP), soluble intercellular adhesion molecule-1 (sICAM-1) and soluble E-selectin (sE-selectin) as well as blood lipids were examined.

**Results:**

Individuals with dyslipidemia had higher levels of hsCRP, sICAM-1 and sE-selectin than those without dyslipidemia (all P values<0.001). Compared to the lowest quartile of inflammatory biomarkers, individuals with the highest quartile were more likely to have dyslipidemia (odds ratio, 95% confidence interval: 3.215, 2.551–4.116 for hsCRP; 1.575, 1.253–1.980 for sICAM-1; 1.495, 1.193–1.873 for sE-selectin). Moreover, hsCRP was associated with all the components of dyslipidemia, whereas, sICAM-1 was not related to high density lipoprotein cholesterol (HDL-c) or triglycerides (TAG). Additionally, sE-selectin was just associated with TAG.

**Conclusion:**

Our study indicated that elevated plasma levels of hsCRP, sICAM-1 and sE-selectin were positively and significantly associated with increased risk of dyslipidemia among Mongolians. However, the associations were not identical for different inflammatory biomarkers with the components of dyslipidemia.

## Introduction

With the remarkable improvement of people’s living standard and associated lifestyle changes in China, dyslipidemia has become more prevalent during the last decades. According to the results from the Chinese National Nutrition and Health Survey in 2002 (CNHS 2002), the prevalence of dyslipidemia in Chinese adults was 18.6% [1]. Dyslipidemia, characterized by alterations in the concentration of one or more lipoproteins in the blood, including elevated total cholesterol (TC), triglycerides (TAG), low density lipoprotein cholesterol (LDL-c) and HDL-c, is a well-established risk factor for atherosclerosis and cardiovascular disease. However, not all the components of dyslipidemia account for the same amount of risk. The role of oxidized LDL (ox-LDL) in the pathogenesis of atherosclerosis has long been described [2], whereas the role of isolated TAG in atherosclerosis is controversial [3], and high-density lipoproteins (HDLs) are known to protect against endothelial lesion development [4].

Meanwhile, atherosclerosis is widely recognized as a chronic inflammatory vessel disorder and the most important single contributor to the growing burden of cardiovascular disease. Clinical and epidemiological studies across different ethnic groups demonstrated that biomarkers of inflammation were associated with atherosclerosis, leading to myocardial infarction (MI) and stroke [5], [6]. The inflammation is initiated by the entrance of cholesterol-rich lipoproteins into the arterial wall and the subsequent absorption by macrophages and finally transformed into foamcells. Accumulating evidence suggests that lipid disorders are strongly related to inflammatory processes [7], [8]. However, to our knowledge, no previous study has examined this association in persons of Mongolian ethnicity. Additionally, there are inconsistencies in the literature regarding the relationships between inflammatory biomarkers and the components of dyslipidemia [13], [18], [21]–[23]. Therefore, the purpose of the present study was to examine the association between inflammation and dyslipidemia as well as its components among Mongolian residents of Inner Mongolia in China.

## Methods

### Study Participants

A cross-sectional study was conducted between 2002 and 2003 in Inner Mongolia, an autonomous region in north China. The methods for selection of study participants and data collection have been described elsewhere [9]. Briefly, individuals aged 20 years and older were recruited from 32 villages in 2 adjacent townships located in Kezuohou Banner and Naiman Banner in Inner Mongolia. The majority (nearly 100%) of local residents were Mongolians who had lived there for many generations and maintained a traditional diet and lifestyle [10]. The study population consisted of both farmers and herdsmen whose diets were high in fat and salt. Out of 3475 eligible people, a total of 2589 individuals chose to participate in the study, the response percentage was 74.50%. Those who did not have a measurement for one or more of the components of dyslipidemia were excluded (n = 45) and a total of 2,544 persons were included in this analysis.

### Ethics Statement

This study was approved by the Institutional Review Board at the Soochow University. Written informed consent was obtained for all study participants.

### Data Collection

A standard questionnaire was used to obtain data on demographic characteristics, lifestyle risk factors, family history of cardiovascular disease and personal medical history by trained staff. Smoking was defined as having smoked at least one cigarette per day for 1 year or more. The information regarding amount and type of alcohol consumed during the past years was collected, and alcohol drinking was defined as consuming at least 50 g distillate spirits (∼50% alcohol concentrate, namely, 25 g alcohol) per day for 1 year or more. Three sitting blood pressure measurements were taken for each participant using a mercury sphygmomanometer according to a standard protocol, and there were 30 s intervals between two BP measurements**.** The mean of these three blood pressure measurements was used in the data analysis. Height and body weight were measured by trained staff using a balance beam scale after subjects removed their shoes and were wearing light clothing. Body mass index (BMI) was calculated as weight in kilograms divided by the square of the height in meters (kg/m^2^). Waist circumference was measured at the level of 1 cm above the umbilicus.

Blood samples were collected in the morning after at last 8 h of fasting. All plasma and serum samples were frozen at −80°C until laboratory testing. Fasting blood was measured using a modified hexokinase enzymatic method. The value of hsCRP was determined by an immunoturbidimetric assay on a Beckman Synchron CX5 Delta Clinical System using commercial reagents. The value of sICAM-1 and sE-selectin was measured by an ELISA assay (R & D Systems, Minneapolis, Minnesota, USA) which employed the quantitative sandwich enzyme immunoassay technique. Concentrations of TC, HDL-c and TAG were assessed enzymatically on a Beckman Synchrony CX5 Delta Clinical System (Beckman Coulter, Fullerton, CA, USA) using commercial reagents, and LDL-c concentration was calculated by means of the Friedewald equation for participants who had less than 400 mg/dL TAG [11]. Dyslipidemia was defined as TC≥6.22 mmol/L and/or TAG≥2.26 mmol/L and/or LDL-c≥4.14 mmol/L and/or HDL-c<1.04 mmol/L and/or having received treatment for dyslipidemia in the previous 2 weeks [12].

### Statistical Analysis

The participants were initially categorized into two mutually exclusive groups with and without dyslipidemia. Descriptive statistics for demographic variables, components of dyslipidemia and inflammatory biomarkers were calculated separately for the two groups. The average levels of continuous variables and the prevalence of categorical variables were calculated for the two groups. Mean and 95% confident interval (95% CI) were used to describe normally distributed variables; if the variables were not normally distributed, median and interquartile ranges were used; frequencies were used to describe categorical variables. Differences between the two groups were tested using *t* test for normally distributed continuous variables; Wilcoxon rank-sum test for variables that did not follow a normal distribution and chi-squared test for categorical variables. Logistic regression was used to examine the association between dyslipidemia and the biomarkers of inflammation, and odds ratio (OR) and 95% CI of dyslipidemia were calculated for higher quartiles of hsCRP, sICAM-1 and sE-selectin compared with the lowest quartile. Finally, multivariable-adjusted mean concentrations of individual components of the dyslipidemia were calculated according to quartiles of hsCRP, sICAM-1 and sE-selectin and linear trends were tested. Gender, age, smoking, drinking, systolic blood pressure, diastolic blood pressure, fast plasma glucose were adjusted in multivariable models. All p values were two-tailed and statistical significance level of 0.05 was used. Statistical analysis was conducted using SAS statistical software (version 9.1).

## Results

The descriptive characteristics of 2,544 study participants according to lipid status are shown in Table 1. Among 2589 participants, total 2544 people with complete data were included in this analysis, 1062 subjects (41.75%) of 2544 people were diagnosed as dyslipidemia. Based on the different component of lipids (TC, TAG, LDL-c, HDL-c), the rate of dyslipidemia were 2.91%, 10.26%, 5.46%, 35.14%, respectively. Compared with those without dyslipidemia, participants with dyslipidemia were more likely to be female, have elevated levels of BMI, waist circumference, systolic blood pressure, diastolic blood pressure and fast plasma glucose, and have a higher rate of family history of cardiovascular disease. There was no significant difference in age, drinking and smoking between the two groups. Individuals with dyslipidemia had higher levels of hsCRP (8.07 vs. 5.13 mg/l), sICAM-1 (346.29 vs. 316.75 ng/ml) and sE-selectin (19.90 vs. 17.80 ng/ml) than those without dyslipidemia (all P values<0.001).

**Table 1:** Baseline Characteristics of 2544 Participants According to Category of Lipoprotein Abnormality.

Factors	Controls	Dyslipidemia	P value
No.	1482	1062	
Age, y	46.56(22.02, 71.10)	46.28(22.49, 70.07)	0.581
BMI, kg/m^2^	21.71(15.40, 28.02)	23.03(15.86, 30.20)	<0.001
WC, cm	79.12(63.30, 95.94)	83.16(62.82, 103.50)	<0.001
SBP, mmHg	122(111,140)	126(115,140)	0.002
DBP, mmHg	80(75,90)	83(78,93)	<0.001
FPG, mmol/L	4.70(4.20,5.30)	5.10(4.50,5.70)	<0.001
Male,%	37.99	44.92	<0.001
Current smoker,%	43.66	45.10	0.469
Alcohol consumption,%	32.05	34.93	0.128
Family history of CVD,%	11.88	14.88	0.027
Components of Dyslipidemia			
TC, mmol/L	3.71(2.08, 5.34)	3.79(0.95, 6.63)	0.110
TAG, mmol/L	0.85(0.61,1.15)	1.20(0.76,2.22)	<0.001
LDL-c, mmol/L	2.12(1.60,2.71)	2.28(1.61,3.19)	<0.001
HDL-c, mmol/L	1.35(0.84, 1.86)	0.92(0.43, 1.41)	<0.001
Inflammatory Biomarkers			
hsCRP, mg/L	5.13(3.60,8.34)	8.07(4.77,15.21)	<0.001
sIcam-1, ng/mL	316.75(129.63, 503.87)	346.29(152.72, 539.82)	<0.001
sE-selectin, ng/mL	17.80(14.13,23.56)	19.90(15.53,26.78)	<0.001

Abbreviations: BMI, body mass index; WC, waist circumference; SBP, systolic blood pressure; DBP, diastolic blood pressure; FPG, fast plasma glucose; CVD, cardiovascular disease; TC, total cholesterol; TAG, triglyceride; LDL-c, low-density lipoprotein–cholesterol; HDL-c, high-density lipoprotein-cholesterol; hsCRP, high sensitivity C-reactive protein; sICAM-1, soluble intercellular adhesionmolecule-1; sE-selectin, soluble E-selectin.

The unadjusted and multivariable-adjusted OR (95% CI) of dyslipidemia according to the quartiles of inflammatory biomarkers are presented in Table 2. Compared to the lowest quartile, the multivariable-adjusted OR (95% CI) of dyslipidemia were significantly higher in the top quartile of hsCRP (3.215, 2.551–4.116), sICAM-1 (1.575, 1.253–1.980) and sE-selectin (1.495, 1.193–1.873) and the linear trends were statistically significant (all p-value for linear trend<0.001).

**Table 2:** Unadjusted and Adjusted Odds Ratios (OR) and 95% Confident Intervals (CI) of Dyslipidemia Associated with Elevated Inflammatory Biomarkers.

Quartiles of Biomarker	Odds ratio (95% CI) of dyslipidemia	
	unadjusted	Multivariable-adjusted
hsCRP (mg/L)		
1 (<3.95)	1.000 (reference)	1.000 (reference)
2 (3.95∼5.94)	0.981(0.772–1.246)	1.049(0.821–1.341)
3 (5.94∼11.38)	1.922(1.527–2.418)	1.826(1.441–2.314)
4 (≥11.38)	3.715(2.945–4.687)	3.215(2.551–4.116)
P value for linear trend	<0.001	<0.001
sICAM-1 (ng/mL)		
1 (<260.28)	1.000 (reference)	1.000 (reference)
2 (260.28∼331.20)	1.049(0.840–1.311)	1.074(0.856–1.348)
3 (331.20∼395.48)	1.302(1.045–1.623)	1.215(0.970–1.523)
4 (≥395.48)	1.909(1.534–2.377)	1.575(1.253–1.980)
P value for linear trend	<0.001	<0.001
sE-selectin (ng/mL)		
1 (<14.78)	1.000 (reference)	1.000 (reference)
2 (14.78∼18.50)	1.090(0.873–1.361)	1.016(0.808–1.277)
3 (18.50∼24.83)	1.345(1.080–1.676)	1.259(1.004–1.578)
4 (≥24.83)	1.589(1.277–1.978)	1.495(1.193–1.873)
P value for linear trend	<0.001	0.001

Multivariable adjustment included gender, age, smoking, drinking, SBP, DBP, FPG.

Table 3 displays the multivariable-adjusted mean levels of each components of dyslipidemia by quartiles of inflammatory biomarkers. hsCRP was associated with all the components of dyslipidemia (p-value for linear trend<0.001 for all components). sICAM-1 had a linear association with LDL-c (p-value = 0.002 for linear trend) and TC (p-value = 0.019 for linear trend). sE-selectin was linearly associated with TAG (p-value<0.001 for linear trend).

**Table 3:** Multivariable-adjusted Mean Levels (95% Confidence Intervals) of The Components of Dyslipidemia According to Quartiles of Inflammatory Biomarkers.

Quartiles of Biomarker		TC(mmol/L)	TAG(mmol/L)	LDL-C(mmol/L)	HDL-C(mmol/L)
hsCRP (mg/L)					
1	(<3.95)	3.47(3.38–3.55)	0.91(0.81–1.00)	2.05(1.97–2.13)	1.23(1.21–1.26)
2	(3.95∼5.94)	3.67(3.58–3.75)	1.01(0.92–1.11)	2.25(2.18–2.33)	1.21(1.18–1.24)
3	(5.94∼11.38)	3.82(3.74–3.90)	1.24(1.15–1.33)	2.42(2.35–2.50)	1.15(1.12–1.17)
4	(≥11.38)	4.01(3.92–4.09)	1.90(1.80–1.10)	2.52(2.44–2.60)	1.10(1.08–1.13)
P value for linear trend		<0.001	<0.001	<0.001	<0.001
sICAM-1 (ng/mL)					
1	(<260.28)	3.71(3.63–3.78)	1.27(1.18–1.36)	2.27(2.20–2.34)	1.18(1.16–1.20)
2	(260.28∼331.20)	3.68(3.59–3.77)	1.24(1.14–1.34)	2.24(2.16–2.32)	1.19(1.16–1.22)
3	(331.20∼395.48)	3.74(3.65–3.82)	1.26(1.17–1.36)	2.30(2.22–2.38)	1.18(1.15–1.21)
4	(≥395.48)	3.84(3.75–3.93)	1.28(1.18–1.38)	2.44(2.35–2.52)	1.15(1.12–1.17)
P value for linear trend		0.019	0.780	0.002	0.054
sE-selectin (ng/mL)					
1	(<14.78)	3.77(3.69–3.85)	1.18(1.09–1.27)	2.35(2.28–2.43)	1.18(1.16–1.20)
2	(14.78∼18.50)	3.69(3.61–3.78)	1.18(1.08–1.28)	2.26(2.18–2.34)	1.19(1.17–1.22)
3	(18.50∼24.83)	3.72(3.64–3.81)	1.24(1.14–1.34)	2.31(2.23–2.39)	1.17(1.14–1.20)
4	(≥24.83)	3.76(3.68–3.85)	1.48(1.38–1.58)	2.31(2.23–2.40)	1.15(1.13–1.18)
P value for linear trend		0.946	<0.001	0.667	0.078

Multivariable adjustment included gender, age, smoking, drinking, SBP, DBP, FPG.

## Discussion

In this cross-sectional study among residents of Inner Mongolia, we found that elevated hsCRP, sICAM-1 and sE-selectin levels were positively and significantly associated with dyslipidemia. Individuals with dyslipidemia had higher levels of hsCRP, sICAM-1 and sE-selectin compared to those without dyslipidemia. In addition, individuals with higher levels of hsCRP, sICAM-1 and sE-selectin were at an increased risk of dyslipidemia compared to those with lower values. However, the above biomarkers demonstrated different profiles with regard to which components of dyslipidimia they were associated with. For example, hsCRP was associated with all the components of dyslipidemia with a dose-response relationship, whereas the positive linear association was only identified between sICAM-1 and LDL-c and TC as well as between sE-selectin and TAG, after adjusting for multivariables.

Our study confirms findings from several previous investigations that have reported a significant association between elevated CRP and dyslipidemia [13], [14]. For example, Martinez-Hervas and Real [13] recently reported that patients with familial combined hyperlipidemia presented with increased levels of xanthine oxidase (XO) activity, serum uric acid, IL-6 and hsCRP compared with healthy controls, suggesting that primary dyslipidemia was an initiator of oxidative stress and inflammation. Therapeutic interventions such as lipid-lowering with statins have been shown to increase flow-mediated dilation (FMD) as well as decrease inflammatory markers, like hsCRP, sICAM-1 or sE-selectin [14]. Furthermore, we explored the relationship between hsCRP and the components of dyslipidemia and found that hsCRP was positively associated with TC, TAG, LDL-c and negatively associated with HDL-c. Alvarez-Sala [15] reported a significant, direct correlation between the relative reduction in hsCRP and the percentage change in levels of LDL-c (r = 0.476; P<0.001) and TC (r = 0.399; P<0.001). Yudkin’s study [16] showed that in 107 nondiabetic subjects, increased plasma concentration of CRP was closely related to the components of insulin resistance syndrome, such as obesity, hypertriglyceridemia and low HDL-c. Another small study [17] including 37 subjects with hypercholesterolemia and 37 controls proved significant associations between hsCRP and TC, HDL-c, LDL-c, ox-LDL, ox-LDL autoantibodies and TAG, which is in close agreement with our study. However, in a study by Moriarty [18], covariance analysis showed that CRP decreases in the atorvastatin group were unrelated to TC or LDL-c reductions; but they were directly related to TAG changes and inversely related to HDL-c changes. IlSIRENTE study [19] demonstrated significant inverse associations of CRP with TC, LDL-c and HDL-c, even after adjustment for potential confounders. However, the mean age (85 years) of the participants in this study is much older than ours (46 years).

Adhesion and transendothelial migration of circulating leukocytes are critical early events in the pathogenesis of atherosclerosis, soluble Cell Adhesion Molecules (sCAMs) such as soluble vascular cellular adhesion molecule-1 (sVCAM-1), sICAM-1 and sE-selectin play pivotal roles in the adhesion of monocytes to endothelial cells. Numerous studies have shown a close association between sCAMs and dyslipidemia [7], [20]. However, the reports on the relationship between sCAMs and the various components of dyslipidemia are disputed. Significant correlations between sICAM-1 and TC (r = 0.13), TAG (r = 0.15), LDL-c (r = 0.19), HDL-c (r = −0.24) were observed in woman in a large cross-sectional study [21], Rohde [22] found that sICAM-1 was positively related to TC and TAG and inversely related to HDL-c. While in Hsu’s [23] study, sICAM-1 was associated with TAG and HDL-c, and no association was found between sICAM-1 and TC or LDL-c. sE-selectin was positively associated with TAG and LDL-c levels and negatively associated with HDL-c levels in three large studies [24]–[26], whereas no association was observed with TC in two of the studies [25], [26]. Some of the smaller studies differ, with some finding a positive association with TAG levels [27] and also TC levels [28] but not with HDL-c level [29]. In present study, we observed significant association between sICAM-1 and LDL-C and TC as well as between sE-selectin and TAG, which indicate that different inflammatory biomarkers may have different mechanisms in the pathogenesis of lipoprotein subclass abnormalities. However, further studies are needed to draw definite conclusions on the relationship between sCAMs and each components of dyslipidemia.

The underlying mechanisms for the risk factor role of inflammation in dyslipidemia are not fully understood. Epidemiological data have indicated that subjects with dyslipidemia are in a proinflammatory state, which is distinguished by elevated levels of cytokines, such as tumor necrosis factor-alpha (TNF-α) and IL-6 and could further promote the expression of hsCRP and CAMs [30]. Particularly, the ongoing inflammation in the artery stimulated by ox-LDL, which leads to the production of cytokines that may induce acute phase proteins such as CRP and sCAMs [31]. There was also evidence to suggest that CRP may have a direct role in atherogenesis by inducing intracellular adhesion molecule expression [5], [32]. Furthermore, both ox-LDL [33] and superoxide anion producted from hypercholesterolemic vessels [34] could inhibit the release of nitric oxide (NO) and down regulate the expression of NO synthase, leading to endothelial dysfunction [35] and the following expression of inflammatory biomarkers [36].

We acknowledge that several limitations of this study should be mentioned. First, this was a cross-sectional study, therefore no causal relationships could be precisely delineated. Secondly, approximately 25% of the eligible population from these villages chose not to participate which may have introduced some selection bias. However, we believe this bias is minimal because it is unlikely that participants chose not to participate due to their lipid levels which they did not know. Furthermore, several important biomarkers of inflammation, such as TNF-α, IL-6 and VCAM-1, were not measured in our study.

In conclusion, our study indicated that elevated plasma levels of hsCRP, sICAM-1 and sE-selectin were positively and significantly associated with increased risk of dyslipidemia among Mongolian residents of Inner Mongolia. However, the associations were not identical for different inflammatory biomarkers with the components of dydlipidemia. Our results suggest that different inflammatory biomarkers may have distinct mechanisms in the pathogenesis of lipoprotein subclass abnormalities.
